# A systematic review of structural brain differences in school-aged children born preterm

**DOI:** 10.1016/j.dcn.2026.101818

**Published:** 2026-09-10

**Authors:** Ebony Coward, Katie Mckinnon, Hilary Richardson, Sue Fletcher-Watson

**Affiliations:** a Salvesen Mindroom Research Centre, University of Edinburgh, United Kingdom; b Department of Child and Adolescent Psychiatry, Institute of Psychiatry, Psychology & Neuroscience, King’s College London, United Kingdom; c Department of Early Life Imaging, School of Biomedical Engineering & Imaging Sciences, King’s College London, United Kingdom; d Department of Psychology, University of Edinburgh, United Kingdom

**Keywords:** Preterm, Childhood, Magnetic resonance imaging, Neurodevelopment

## Abstract

Preterm birth (<37 weeks’ gestation) is commonly associated with adverse developmental outcomes, many of which correlate with structural brain differences. Differences in brain structure exist between preterm and term-born neonates/babies at term-equivalent age; however, it remains to be determined whether these differences persist in childhood. Two databases, EMBASE and Ovid MEDLINE, were searched to identify all publications using magnetic resonance imaging (MRI) to measure differences in brain structure between preterm and term-born children at 6–10 years old and associated perinatal risk factors. A risk of bias assessment was performed using the ROBINS-E tool. 23 articles were included that reported MRI data from prospective, longitudinal cohort studies. Preterm children showed smaller global and regional tissue volumes and altered white matter microstructure compared to term-born controls. These associations were more pronounced for those born at a lower gestational age and in the presence of perinatal brain injury. Across studies, structural brain differences had varied associations with neurodevelopmental outcomes. Furthermore, this review highlights that there is a clear lack of prospective, longitudinal studies reporting on the association between preterm birth and MRI differences at school age.

## Highlights

• Preterm birth is associated with structural brain differences in school-aged children.
• MRI studies show reduced brain tissue volumes and altered white matter microstructure.
• Gestational age at birth and the presence of perinatal brain injury contribute to the neuroanatomical differences.

## Graphical Abstract

**Figure:**
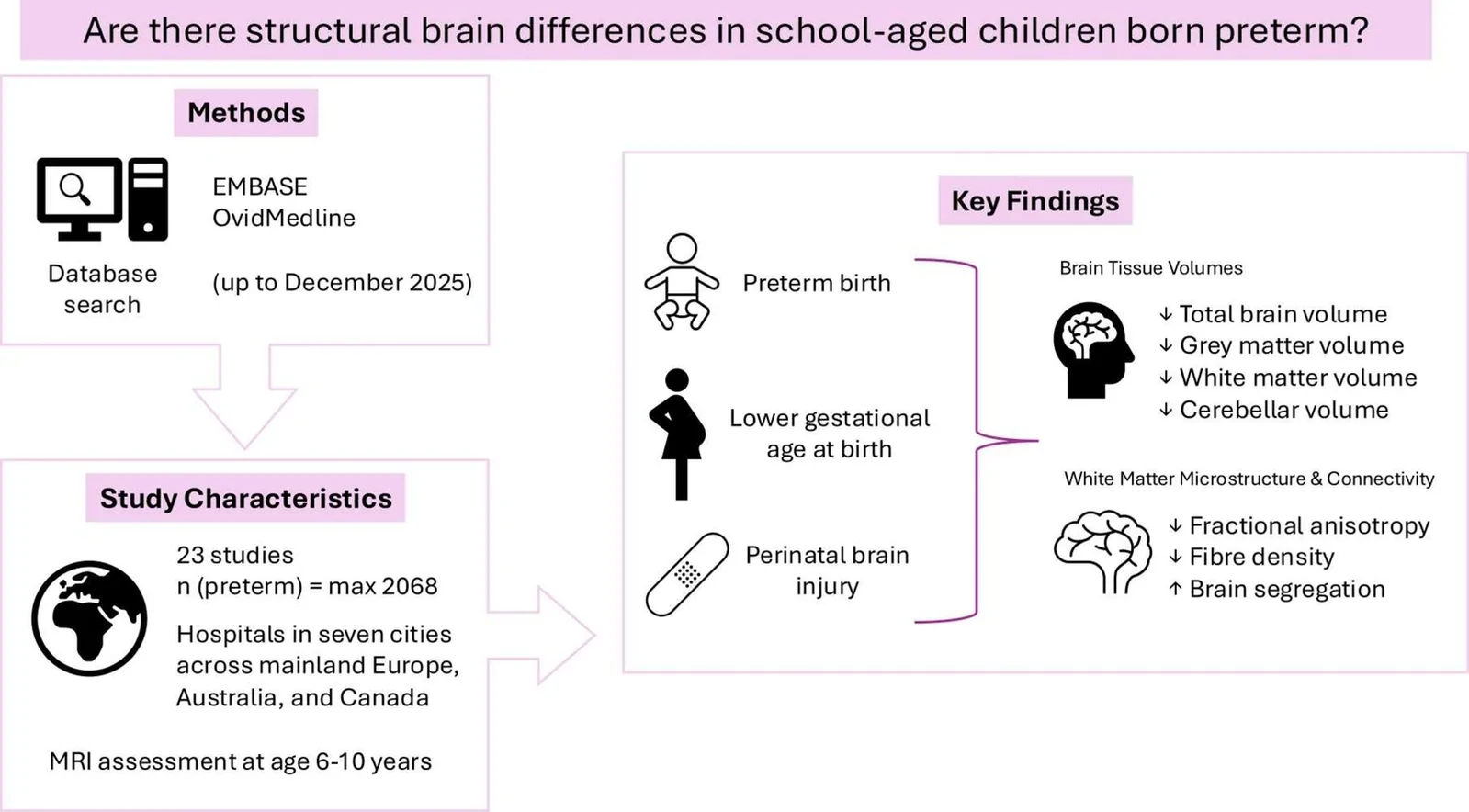

## 1 Introduction

Preterm birth is commonly defined as a delivery that occurs before 37 weeks’ gestation (WHO, 2012). Although the past 30 years have seen significant advances in perinatal care, preterm birth still carries an increased risk of adverse outcomes, including chronic disease, sensorimotor impairments, learning difficulties, and behavioural disorders (Johnson and Marlow, 2017, Ream and Lehwald, 2018). A retrospective cohort study of over 400,000 children in Scotland found that a lower gestational age (GA) at birth was associated with an increased risk of special educational needs (SEN) at school age (MacKay et al., 2010) and there is a wealth of evidence for more specific atypical cognitive outcomes in preterm born children (Anderson, 2014, Brydges et al., 2018, Orchinik et al., 2011, Pritchard et al., 2009, Woodward et al., 2009). The likelihood of neurodevelopmental challenges increases with earlier GA, with moderate to severe developmental delay present at two years in 12–21% of infants born at < 30 weeks (Blankenstein et al., 2023). Given the high rates of neurodevelopmental difficulty in later life, researchers have sought neuroanatomical causes.

Early-life imaging studies have allowed researchers to investigate the impact of prematurity on the developing brain by using a variety of techniques ranging from conventional magnetic resonance imaging (MRI), which measures brain structure (e.g., cortical volume/surface area), to diffusion tensor imaging (DTI), which measures white-matter connectivity (Hinojosa-Rodríguez et al., 2017, Raffelt et al., 2017). DTI metrics of white matter microstructure represent myelination and white matter maturation, key features of encephalopathy of prematurity (Inder et al., 2023). Common MRI measures and terminology used in early-life imaging studies are described in the Glossary.

Early-life imaging studies often involve participants from different stages of prematurity, i.e., moderate to late preterm (32–36 weeks gestation), very preterm (28–32 weeks gestation) and extremely preterm (less than 28 weeks gestation) (WHO, 2012). Evidence suggests that there are significant whole brain differences between preterm and term-born children in the early neonatal period. Primarily, preterm infants show smaller white and grey matter volumes, abnormal cortical folding, primarily lower gyrification index, and reduced cortical maturation (Boardman et al., 2006, Engelhardt et al., 2015, Estep et al., 2014, Thompson et al., 2019, Viola et al., 2011). This is further exacerbated by perinatal brain injury – a common consequence of preterm birth resulting in white matter abnormalities such as lesions, loss of volume and delayed myelination (Anderson et al., 2015). It is likely that these differences arise due to the disruption of active neurodevelopment, as most preterm births occur in the third trimester where rapid neuron proliferation, synaptogenesis, and myelination occur (Huang et al., 2006, Kostović and Jovanov-Milošević, 2006). Preterm birth is associated with lower fractional anisotropy (FA) and higher mean diffusivity (MD) (Barnett et al., 2018). Regions of higher volume and maturation have also been observed in preterm infants, particularly in the sensory and motor cortices (Alexander et al., 2019, Thompson et al., 2019, Viola et al., 2011). These differences may be a consequence of early exposure to the extrauterine environment, unrestricted motor movement, and sensory stimuli in the neonatal intensive care unit (NICU) (Alexander et al., 2019, Viola et al., 2011). Early atypical brain structure and connectivity is therefore a common consequence of preterm birth and can have long-term associations with cognitive development (Keunen et al., 2012, Moore et al., 2013). Structural and functional networks begin to develop before term, and although many key network hubs remain intact following preterm birth, the development of specific connections can be affected (Ball et al., 2014, Batalle et al., 2017, Oldham and Fornito, 2019).

Studies have also shown that there are long-term differences in brain structure and connectivity in both adolescence and adulthood. Preterm-born participants show reduced total brain volume, localised reductions in white matter and grey matter volumes, and altered connectivity compared to age-matched term-born controls, particularly with younger GA (deKieviet et al., 2012, Kelly et al., 2023, Kontis et al., 2009, Meng et al., 2016, Nosarti et al., 2014, Nosarti et al., 2002).

However, there is less evidence investigating the potential impact in middle childhood, particularly at the start of formal education, perhaps given the difficulties inherent to acquiring MRI data with children. MRI evidence from children could provide direct insight into the neurocognitive mechanisms of behavioural differences observed during this important time of life, when access to extra support and/or targeted interventions could have a significant impact on future outcomes. The purposes of this review were (1) to investigate whether whole brain differences, as measured via MRI, exist between preterm and term-born children at primary school age (6–10 years), (2) to establish evidence of perinatal risk or protective factors that influence brain differences, and (3) to investigate whether brain differences relate to outcomes.

## 2 Methods

A systematic literature search was conducted to identify MRI studies of preterm cohorts at school age. Eligible studies were identified using EMBASE (1974–15 December 2025) and Ovid MEDLINE (1946–15 December 2025). Search terms encompassed the following keywords and a range of synonyms: ‘preterm birth’, ‘magnetic resonance imaging’, ‘child’, ‘prospective study’, ‘brain structure’, ‘volume’ and ‘connectivity’. The full search strategy is described in the Supplementary Material.

### 2.1 Inclusion and exclusion criteria

Studies were included if they reported on a preterm cohort born before 37 weeks’ gestation and had completed at least one MRI scan between the ages of 6–10 years. The search was limited to studies published after 2006 in order to capture a contemporary cohort born in the year 2000 or later, when hallmarks of perinatal care such as antenatal corticosteroids and surfactant therapy have become largely routine (Polin et al., 2014). Improvements in neonatal care have reduced mortality for preterm infants, and although evidence for improvements in cognitive, educational, and behavioural outcomes remains sparse, there have been reductions in cerebral palsy and rates of white matter injury (Cheong et al., 2020, Selvanathan et al., 2024, Twilhaar et al., 2018).

Studies were excluded from the review if they met any of the following criteria: retrospective study design; no term-born control group; cohort born exclusively before the year 2000; highly selective preterm cohort, e.g., only including children with perinatal brain injury; only functional MRI data reported; no original clinical data reported.

### 2.2 Risk of bias assessment

The Risk of Bias in Non-Randomised Studies of Exposures (ROBINS-E) tool was used to assess bias in each of the studies included. This is a relatively new tool that has been adapted from the ROBINS-I tool for intervention studies. ROBINS-E divides the risk of bias assessment into seven domains: confounders, participant recruitment and selection, classification of exposures, coexposures, missing data, measurement of outcomes and selection of the reported result (Higgins et al., 2024). For each of the domains a risk of bias rating is assigned as high, moderate, or low and an overall bias judgement is derived.

### 2.3 Screening

**Fig. 1:**
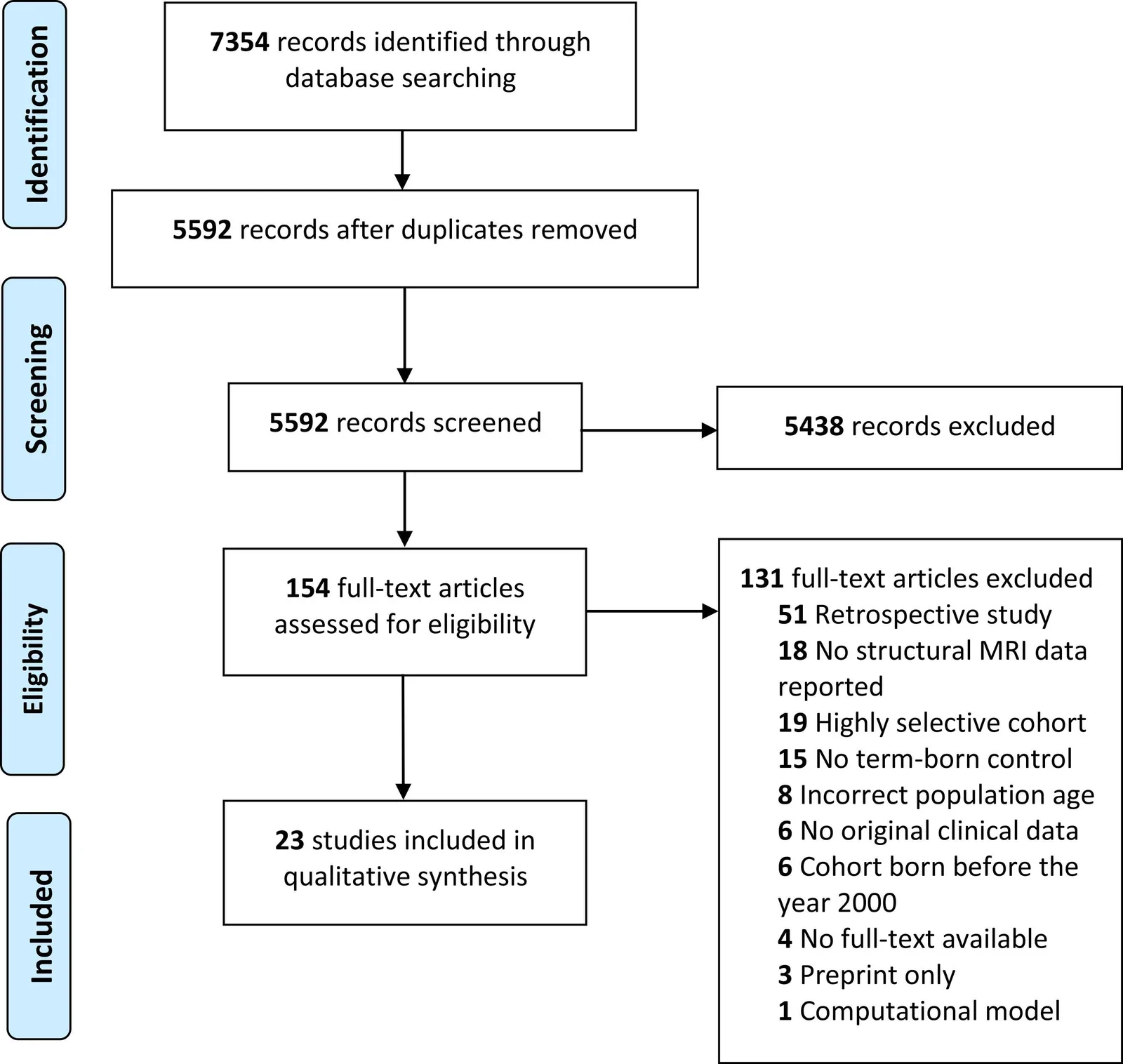
PRISMA flow diagram showing the literature screening process and selection of eligible studies included in the review (Moher et al., 2009).

The initial database search produced 7354 records and after removing duplicates, 5592 records were imported into EndNote for title and abstract screening, as described in the PRISMA flow chart (Fig. 1). Following this, 154 full-text articles were retrieved and assessed for eligibility. Of these, 131 studies were excluded based on specific exclusion criteria, and the 23 remaining studies were included in the review. Screening was performed by two authors (EC and KM), with discrepancies discussed and resolved by an additional team member as required.

## 3 Results

A summary of all 23 studies included in the review is provided in Table 1. The studies report data on preterm cohorts recruited from hospitals in seven cities across mainland Europe, Australia, and Canada. In total, there were up to 1801 preterm participants, however, there is likely some overlap between cohorts derived from the same hospital. For instance, a large observational study at the Royal Women’s Hospital in Australia yielded ten papers (Kelly et al., 2022, Kelly et al., 2024, Kelly et al., 2021, Kelly et al., 2020, Kelly et al., 2016, Monson et al., 2016, Thompson et al., 2022, Thompson et al., 2020, Thompson et al., 2016, Zhang et al., 2015), however it is not possible to identify whether each participant is unique. All studies had a prospective design with hospital-based preterm cohorts recruited from birth.

**Table 1:** A summary of the main characteristics and MRI findings of all studies included in the review.

**Study and location**	**Cohort characteristics**		**Participant inclusion criteria**	**MRI field strength (Tesla/T) and motion correction method**	**Brain measures**	**Findings for preterm-born relative to term-born children**	**Perinatal factor or neurodevelopmental outcome**
(Broström et al., 2025)*** Stockholm, Sweden	Cohort size (n)	EPT: 42 T: 25	Birth < 27 weeks’ GA. No cerebral palsy.	3.0 T Volumes were manually inspected and excluded if poor quality.	Brain tissue volume (adjusted for ICV)	Smaller volumes of the thalami, basal ganglia, cerebellum, motor execution network (precentral and postcentral gyri) and motor imagery network (precuneus, supplementary motor area, superior frontal gyri, precentral gyri).	Children born preterm with motor impairment (aiming and catching difficulties) at 12 years, compared to preterm infants without motor impairment, had smaller volumes in the basal ganglia, motor execution network, motor imagery network, and cerebellum.
	Sex (%)	M: 49.3 F: 50.7					
	Mean GA at birth (weeks)	EPT: 25.6 T: 40.0					
	Mean age at MRI (years)	EPT: 9.9 T: 10.2					
(deKieviet et al., 2014) VU University Medical Center (Amsterdam, Netherlands)	Cohort size (n)	VPT: 30 T: 47	No major congenital or chromosomal anomalies, no transfer to another hospital < 48 h after birth or admission from an extra-regional hospital.	1.5 T	Brain tissue volume (adjusted for TBV) White matter microstructure (FA)	Smaller GM, WM, cerebellum, thalamus, hippocampus, striatum. Lower values for 18 white matter tracts, including: cingulum hippocampal tract, corticospinal tract, forceps major, forceps minor, inferior fronto-occipital tract (left). No difference on excluding those with brain injury.	Reduced FA correlated with motor impairment.
	Sex (%)	M: 43.3 F: 56.7		FSL eddy current and motion correction; visual screening and removal of volumes with artifacts.			
	Mean GA at birth (weeks)	VPT: 28.9 T: > 37					
	Mean age at MRI (years)	VPT: 8.6 T: 8.7					
(deKieviet et al., 2021) VU University Medical Center (Amsterdam, Netherlands)	Cohort size (n)	VPT: 29 T: 52	No major congenital or chromosomal anomalies, no transfer to another hospital < 48 h after birth or admission from an extra-regional hospital.	1.5 T	White matter connectome	Higher degree of brain segregation, no significant difference in integration, higher small-worldness.	Small-worldness correlatied with motor outcomes.
	Sex (%)	M: 41.4 F: 58.6		FSL eddy current and motion correction; visual screening and removal of volumes with artifacts			
	Mean GA at birth (weeks)	VPT: 28.9 T: > 37					
	Mean age at MRI (years)	VPT: 8.6 FT: 8.7					
(ElMarroun et al., 2020) Rotterdam, Netherlands	Cohort size (n)	PT: 138 T: 2718	No twin pregnancies.	3.0 T	Brain tissue volume (adjusted for TBV)	Smaller TBV, GM and cerebellar WM volumes. Positive association between GA and brain volumes. Full adjustment including for maternal education gave similar associations.	N/A
	Sex (%)	M: 49.8 F: 50.2		FreeSurfer reconstructions were visually inspected; exclusion of images not suitable for analysis			
	Mean GA at birth (weeks)	PT: 34.6 T: 40.0					
	Mean age at MRI (years)	PT: 10.2 T: 10.1					
(FernándezDeGamarra-Oca et al., 2024)*** Stockholm, Sweden	Cohort size (n)	EPT: 51 T: 40	Birth < 27 weeks’ GA. No severe white matter abnormalities or abnormal ventricular shapes not suggestive of white matter abnormalities.	3.0 T Not specified	Cortical thickness	Thinner mean cortex. Thinner regional cortex of these bilateral areas: banks of the superior temporal sulci, caudal middle frontal, fusiform, inferior parietal, medial orbitofrontal, middle temporal, pars orbitalis, posterior cingulate, precentral, precuneus, superior parietal, superior temporal, supramarginal, temporal pole, and insula. Additionally, thinner regional cortex of these unilateral areas: left inferior temporal, right lateral orbital, left pas opercularis, left pars triangularis, left pericalcarine.	Discrete white matter abnormalities in preterm infants were associated with thicker bilateral mean cortex, and regional differences in cortical thickness. Thicker: bilateral (caudal middle frontal, precentral, pars orbitalis, rostral middle frontal, superior frontal), left (lateral orbitofrontal, cuneus, paracentral, pars opercularis, insula) and right (inferior temporal, middle temporal, pars triangularis, superior temporal gyri, temporal pole). Thinner: left caudal anterior cingulate gyrus. No differences in global cortical volume. No correlation between cortical volume or bilateral mean cortical thickness with fine or gross motor function.
	Sex (%)	M: 49.5 F: 50.5					
	Mean GA at birth (weeks)	EPT: 25.4–25.8 T: Term (not clear)					
	Mean age at MRI (years)	EPT: 10.3 T: 10.1					
(Kelly et al., 2016)* Royal Women’s Hospital (Melbourne, Australia)	Cohort size (n)	EPT: 145 FT: 33	No major congenital or chromosomal anomalies.	3.0 T	White matter microstructure (FA, NODDI)	Lower FA and higher axon dispersion in right cingulum (hippocampal part), right inferior longitudinal fasciculus, right inferior fronto-occipital fasciculus, right uncinate fasciculus, right anterior thalamic radiation, right external capsule.	Low FA, high axon dispersion, and low axon density correlate with poor neurodevelopmental outcomes.
	Sex (%)	M: 49.0 F: 51.0		FSL eddy current and motion correction			
	Mean GA at birth (weeks)	EPT: 27.5 T: 38.9					
	Mean age at MRI (years)	EPT: 7.5 T: 7.6					
(Kelly et al., 2020)* Royal Women’s Hospital (Melbourne, Australia)	Cohort size (n)	EPT: 138 T: 45	No major congenital or chromosomal anomalies.	3.0 T	White matter microstructure (FD, FC, FDC) (adjusted for ICV)	Smaller values mostly in corpus callosum, tapetum, inferior fronto-occipital fasciculus, fornix, and cingulum. FD was 21% lower on average. FC and FDC group differences not significant after adjusting for intracranial volume.	Low birthweight z-score associated with low FC and FDC across the white matter, although not after adjusting for ICV.
	Sex (%)	M: 49.0 F: 51.0		FSL eddy current and motion correction			
	Mean GA at birth (weeks)	EPT: 27.5 T: 38.9					
	Mean age at MRI (years)	1st MRI: EPT: 0 T: 0 2nd MRI: EPT: 7.5 T: 7.6 3rd MRI: EPT: 13.3 T: 13.3					
(Kelly et al., 2021)* Royal Women’s Hospital (Melbourne, Australia)	Cohort size (n)	VPT: 144 T: 33	No major congenital or chromosomal anomalies.	3.0 T FSL eddy current and motion correction Volumes were manually inspected and excluded if poor quality.	Brain tissue volumes White matter microstructure (FA, MD, AD, RD, neurite orientation dispersion neurite density)	Lower volume of the fornices bilaterally, and higher volume in anterior thalamic radiation. Higher MD, AD, RD, and neurite orientation dispersion of the fornices bilaterally. Lower neurite density in left fornix. Higher neurite orientation dispersion in left superior-lateral medial forebrain bundle. No significant differences in cingulums or uncinate fasciculi.	Higher FA and lower MD/AD/RD in cingulum, uncinate fasciculus, medial forebrain bundle, and anterior thalamic radiation associated with better episodic memory.
	Sex (%)	M: 48.6 F: 51.4					
	Mean GA at birth (weeks)	VPT: 27.5 T: 38.9					
	Mean age at MRI (years)	VPT: 7.5 T: 7.6					
(Kelly et al., 2022)* Royal Women’s Hospital (Melbourne, Australia)	Cohort size (n)	VPT: 120 T: 29	No major congenital or chromosomal anomalies.	3.0 T FSL eddy current and motion correction Volumes were manually inspected and excluded if poor quality.	Brain tissue volumes Brain age delta based on regional volume, area, and thickness	Smaller intracranial volume. No significant difference in average brain age delta (difference between predicted and actual age). No significant difference in variance of brain age delta at 7 years, but was seen at 13 years.	No association between brain age delta and neurodevelopmental outcomes (IQ, memory, language and motor scores).
	Sex (%)	M: 47.7 F: 52.3					
	Mean GA at birth (weeks)	VPT: 27.4 T: 38.9					
	Mean age at MRI (years)	1st MRI: VPT: 7.5 T: 7.6 2nd MRI: VPT: 13.3 T: 13.3					
(Kelly et al., 2024)* Royal Women’s Hospital (Melbourne, Australia)	Cohort size (n)	VPT: 201 T: 66	No major congenital or chromosomal anomalies.	3.0 T FSL eddy current and motion correction Volumes were manually inspected and excluded if poor quality.	Brain tissue volumes Cortical surface area Cortical thickness	Reduced cortical volumes at 0 years and lower rates of increase in cortical volumes between 0 and 7 years, with greater magnitude of volume reductions at 7 years for the middle temporal, supramarginal, pars opercularis, pars triangularis, medial orbitofrontal, and insula regions. Cortical area reductions at 0 years, with most areas showing no difference in area trajectories between 0 and 7 years. Increased reductions in cortical area trajectories between 0 and 7 years the inferior orbital frontal regions. In many regions, thicker cortex at 0 years, with smaller increases between 0 and 7 years, so similar or thinner cortex at 7 years (lingual, cuneus, precuneus, inferior parietal, fusiform, precentral, pars opercularis, pars triangularis, superior frontal regions).	Smaller increases in cortical thickness 0–7 years in pars opercularis, precuneus, paracentral, and lingual regions in children with impaired language. Smaller increases in cortical thickness 0–7 years in precuneus, precentral, paracentral, and linugal regions in children with impaired memory. Smaller increase in cortical volume of the left pars opercularis in children with impaired language.
	Sex (%)	M: 52.2 F: 47.8					
	Mean GA at birth (weeks)	VPT: 27.5 T: 39.1					
	Mean age at MRI (years)	1st MRI: VPT: 0 T: 0 2nd MRI: VPT: 7.5 T: 7.6 3rd MRI: VPT: 13.3 T: 13.3					
(Kvanta et al., 2021)*** Stockholm, Sweden	Cohort size (n)	EPT: 51 T: 38	Birth < 27 weeks’ GA. No severe medical conditions, congenital or chromosomal abnormalities, or major brain lesions.	3.0 T Volumes were manually inspected and excluded if poor quality.	Brain tissue volume (adjusted for ICV)	Larger GM volumes, smaller WM volumes, smaller cerebral parenchymal volumes, no difference in CSF volumes.	N/A
	Sex (%)	M: 48.3 F: 51.7					
	Mean GA at birth (weeks)	EPT: 25.6 T: 40.1					
	Mean age at MRI (years)	EPT: 10.3 T: 10.1					
(Kvanta et al., 2023)*** Stockholm, Sweden	Cohort size (n)	EPT: 51 T: 38	Birth < 27 weeks’ GA. No severe medical conditions, congenital or chromosomal abnormalities, or major brain lesions.	3.0 T Volumes were manually inspected and excluded if poor quality.	Brain tissue volume (adjusted for ICV)	Smaller GM and WM in temporal lobes. Smaller GM in precuneus gyri. Smaller WM in anterior cingulum. Larger GM and WM in right posterior cingulum, and occipital lobes.	Children with IVH grades I-II compared with no IVH had smaller GM in left hippocampus. Children with PDA requiring ligation compared with no PDA treatment had smaller WM in left frontal lobe, and larger GM in occipital lobes. Children with PDA requiring ligation compared with ibuprofen had larger GM in occipital lobes. No regional volume differences between children with PDA treated with ibuprofen and no PDA.
	Sex (%)	M: 48.3 F: 51.7					
	Mean GA at birth (weeks)	EPT: 25.6 T: 40.1					
	Mean age at MRI (years)	EPT: 10.3 T: 10.1					
(Kvanta et al., 2024)*** Stockholm, Sweden	Cohort size (n)	EPT: 50 T: 37	Birth < 27 weeks’ GA. No severe medical conditions or brain lesions.	3.0 T Volumes were manually inspected and excluded if poor quality.	Brain tissue volume (adjusted for ICV)	Smaller regional brain volumes across all language-related regions except the left Heschl’s gyrus (supplementary motor area, inferior frontal gyrus triangular/opercular, Heschl’s gyrus, angular gyrus, supramarginal gyrus, superior/middle/inferior temporal gyri, summed language regions). No significant brain asymmetry. Thinner regional cortex across most language-related regions (right inferior frontal gyrus triangular/opercular, bilateral inferior parietal gyrus, superior/middle temporal gyri, left inferior temporal gyrus).	In preterm infants, volume of the summed language regions positively correlated with vocabulary and recalling sentences scores. In term infants, cortical thickness in the inferior frontal gyrus and opercular part negatively correlated with recalling sentences scores. These results did not survive adjustment for IQ.
	Sex (%)	M: 47.1 F: 52.9					
	Mean GA at birth (weeks)	EPT: 25.6 T: 40.0					
	Mean age at MRI (years)	EPT: 10.3 T: 10.2					
(Kvanta et al., 2025)*** Stockholm, Sweden	Cohort size (n)	EPT: 42 T: 29	Birth < 27 weeks’ GA. No severe medical conditions or severe brain abnormalities.	3.0 T Volumes were manually inspected and excluded if poor quality.	Brain tissue volume (adjusted for ICV) Cortical thickness	Smaller mean GM and WM volumes and ICV. Thinner mean cortex bilaterally.	In the whole cohort, GM and WM volume positively correlated with IQ at 12 years. In preterm infants, GM volume in bilateral insulae positively correlated with IQ. In preterm infants, no correlation between brain volumes in the neonatal period, or longitudinal change in brain volumes between the neonatal period and 10 years, with IQ. Mean right cortical thickness negatively correlated with IQ and visual spatial index, although not after correction for multiple comparisons.
	Sex (%)	M: 47.9 F: 52.1					
	Mean GA at birth (weeks)	EPT: 25.6 T: 40.0					
	Mean age at MRI (years)	EPT: 10.5 T: 10.3					
(Monson et al., 2016)* Royal Women’s Hospital (Melbourne, Australia)	Cohort size (n)	EPT: 134 T: 33	No major congenital or chromosomal anomalies.	3.0 T Not specified.	Brain tissue volume (adjusted for ICV)	Smaller GM volumes. No evidence of a relationship between PT birth and GA at birth, WMI score, SGA, BPD, or infection.	Volumes correlated with cognition, language, and motor functioning.
	Sex (%)	M: 50.0 F: 50.0					
	Mean GA at birth (weeks)	EPT: 27.5 T: 38.9					
	Mean age at MRI (years)	EPT: 7.5 T: 7.6					
(Thompson et al., 2016)* Royal Women’s Hospital (Melbourne, Australia)	Cohort size (n)	EPT: 107 T: 26	Participants with and without perinatal complications.	3.0 T	White matter connectivity (adjusted for ICV)	Lower density connections, reduced global efficiency, and higher local efficiency. No significant difference in small-worldness.	Infection correlated with reduced connectivity. Reduced connectivity associated with cognition and motor impairment.
	Sex (%)	M: 43.0 F: 57.0		FSL eddy current and motion correction			
	Mean GA at birth (weeks)	EPT: 27.4 T: 38.8					
	Mean age at MRI (years)	EPT: 7.5 T: 7.6					
(Thompson et al., 2020)* Royal Women’s Hospital (Melbourne, Australia)	Cohort size (n)	EPT: 152 T: 34	No major congenital or chromosomal anomalies.	3.0 T	Brain tissue volume (adjusted for TBV)	No statistically significant differences in volumetric growth between ages 7 and 13, before or after adjusting for TBV. No association between moderate to severe brain abnormality and volumetric growth between 7 and 13 years, before or after adjusting for TBV. No association between birthweight and volumetric growth before adjusting for TBV. After adjusting for TBV, poorer growth in left insula associated with being born SGA. BPD not associated with volumetric growth between ages 7–13 before or after adjusting for TBV.	Brain injury associated with poor growth 0–7 y. Socioeconomic factors and bronchopulmonary dysplasia did not correlate.
	Sex (%)	M: 52.0 F: 48.0		Volumes were manually inspected and excluded if poor quality.			
	Mean GA at birth (weeks)	EPT: 27.5 T: 39.0					
	Mean age at MRI (years)	1st MRI: EPT: 0 T: 0 2nd MRI: EPT: 7.5 T: 7.6 3rd MRI: EPT: 13.3 T: 13.4					
(Thompson et al., 2022)* Royal Women’s Hospital (Melbourne, Australia)	Cohort size (n)	VPT: 224 T: 76	No major congenital or chromosomal anomalies.	3.0 T FSL eddy current and motion correction Volumes were manually inspected and excluded if poor quality.	White matter microstructure (T1w/T2w)	Lower T1w/T2w values in bilateral cingula (cingular part).	Being SGA associated with lower T1w/T2w values in many regions, and with less increase in T1w/T2w 0–7 years. No association with postnatal infection or BPD. Faster increase in T1w/T2w values 7–13 years associated with higher IQ for all children (mainly left uncinate fasciculus). Change in T1w/T2w 0–7 years in various regions associated with attention, executive function, and mathematics in term-born children only.
	Sex (%)	M:51.2 F: 48.8					
	Mean GA at birth (weeks)	VPT: 27.5 T: 39.1					
	Mean age at MRI (years)	1st MRI: VPT: 0 T: 0 2nd MRI: VPT: 7.5 T: 7.6 3rd MRI: VPT: 13.3 T: 13.2					
(Tokariev et al., 2019) Helsinki University Central Hospital (Helsinki, Finland)	Cohort size (n)	EPT: 16 T: 16	No WMI, chromosomal abnormality, global cognitive impairment, CP, ADHD, insufficient native language skills, mutism, or metal in body.	3.0 T	White matter microstructure (FA, MD, RD, AD)	Larger FA values in superior longitudinal fasciculus, inferior fronto-occipital fasciculus/inferior longitudinal fasciculus, cortico-spinal tract, cerebellar WM. No regions where FA was greater in FT than EPT. Lower MD, RD, and AD in EPT children.	High FA associated with poor visuospatial scores.
	Sex (%)	M: 56.0 F: 44.0		TORTOISE eddy current and motion correction			
	Mean GA at birth (weeks)	EPT: 26.7 T: 40.0					
	Mean age at MRI (years)	EPT: 7.5 T: 7.6					
(Vandewouw et al., 2019)** Hospital for Sick Children (Toronto, Canada)	Cohort size (n)	VPT: 120 T: 146	No major congenital or chromosomal anomalies.	3.0 T	Brain tissue volume Cortical thickness Cortical surface area	Smaller TBV, %WM (increases more with age). Larger % volume of striatum. No correlation between GA and TBV, % GM or %WM volume. Reduced in bilateral midcingulate cortex but higher in bilateral occipital and orbitofrontal cortices, the bilateral inferior temporal and fusiform gyri, and the right inferior pre-and post-central gyri. Decreases more with age. Positively correlated with age in right precuneus, cingulate cortex and temporal gyri. Smaller in bilateral posterior part of the fusiform gyrus, the left precuneus, superior parietal and orbitofrontal gyri, and gyrus rectus, and the right middle temporal, posterior and middle cingulate gyri. Larger values in bilateral inferior and middle temporal gyri, superior temporal pole, parahippocampal and anterior fusiform gyri, and the right superior temporal, superior and middle frontal gyri. Negative correlation with GA.	N/A
	Sex (%)	M: 54.0 F: 46.0		Visually inspected brain masks; excluded images due to registration/segmentation failure.			
	Mean GA at birth (weeks)	VPT: 28.26 T: > 37					
	Mean age at MRI (years)	VPT: 6.76 T: 7.17					
(Young et al., 2019)** Hospital for Sick Children (Toronto, Canada)	Cohort size (n)	VPT: 23 T: 24	No major congenital or chromosomal anomalies.	3.0 T	White matter microstructure (FA, MD, RD, NODDI)	Lower FA in corpus callosum, anterior and posterior limb of the internal capsule, corona radiata (anterior, posterior, and superior), external capsule, posterior thalamic radiation, and cingulum. Higher MD, RD and ODI. Early white matter injury associated with lower ODI in right superior corona radiata. No significant association with germinal matrix/ intraventricular haemorrhage.	High NDI/ODI correlated with low IQ. Brain injury associated with low ODI.
	Sex (%)	M: 69.6 F: 30.4		FSL eddy current and motion correction; visual screening for gross motion artifacts.			
	Mean GA at birth (weeks)	VPT: 28.8 T: > 37					
	Mean age at MRI (years)	VPT: 6.57 T: 6.62					
(Zhang et al., 2015)* Royal Women's Hospital (Melbourne, Australia)	Cohort size (n)	EPT: 24 T: 24	No severe WMI or SGA.	3.0 T	Brain tissue volume Cortical surface area	Smaller WM and GM volume in lateral parietal cortex, orbital frontal cortex, middle temporal cortex, and insular cortex of both hemispheres, inferior frontal region of the left hemisphere and postcentral region of the right hemisphere. Larger GM in lateral occipital lobe of the left hemisphere and medial parietal lobe of the right hemisphere. Approx. 10% smaller. Relative cortical surface area smaller in posterior superior temporal and angular gyri bilaterally, the inferior precentral and inferior postcentral cortex in the left hemisphere, and the ventral sensorimotor cortex in the right hemisphere. Larger values in medial frontoparietal cortex.	N/A
	Sex (%)	M: 50.0 F: 50.0		Not specified.			
	Mean GA at birth (weeks)	EPT: 25.0 T: 39.0					
	Mean age at MRI (years)	EPT: 7.4 T: 7.4					
(Zubiaurre-Elorza et al., 2012) Hospital Universitari Vall d’Hebron (Barcelona, Spain)	Cohort size (n)	PVLVPT: 22 VPT: 14 T: 22	PVL-PT: Full intelligence quotient (FIQ) > 70, neonatal diagnosis of PVL, and signs of PVL in MRI scan, no IVH grades III/IV, or other severe perinatal brain injury. PT: birth weight below 2500 g, APGAR score at 5 mins > 7, no major perinatal complications, no severe brain injury, no observable pathology on neonatal cranial ultrasound.	3.0 T	Cortical thickness	Thinner in VPT than in FT in left postcentral, supramarginal, and caudal middle rostral gyri. Thicker in PVL-VPT than in FT in left lateral occipital area and right cuneus but thinner in right middle temporal and right lingual regions. Thicker in PVL-VPT than in VPT in left rostral middle frontal area and right pericalcarine area.	Thicker cortex associated with brain injury. Regional thickness positively and negatively associated with internalising and externalising scores.
	Sex (%)	M: 63.9 F: 36.1		Not specified.			
	Mean GA at birth (weeks)	PVLVPT: 30.2 VPT: 31.9 T: 39.5					
	Mean age at MRI (years)	PVL-VPT: 8.7 VPT: 9.2 T: 9.3					

Cohorts were broadly classified as preterm (PT), but also more specifically as very preterm (VPT) or extremely preterm (EPT) based on gestational age (GA) at birth. One study (Zubiaurre-Elorza et al., 2012) included a VPT cohort with periventricular leukomalacia (PVL) in addition to a group without PVL. Each study had a term (T) control group (≥37 weeks’ GA). Cohort size includes all participants who provided usable MRI data. Global brain tissue volumes were measured in cm3 and were compared between groups, looking specifically at differences in total brain volume (TBV), grey matter (GM) and white matter (WM) volumes. Differences in white matter microstructure were assessed using a range of metrics including fractional anisotropy (FA), mean diffusivity (MD), axial diffusivity (AD), radial diffusivity (RD), fibre density (FD), fibre cross section (FC), combined fibre density and cross section (FDC), and neurite orientation, density, and dispersion (NODDI). White matter connectivity was assessed by measuring brain segregation, integration and small-worldness. Several studies provided data on the impact of perinatal factors, e.g., white matter injury (WMI), bronchopulmonary dysplasia (BPD), and small for gestational age (SGA). In their analyses, some studies included an adjustment for TBV (deKieviet et al., 2014, ElMarroun et al., 2020, Thompson et al., 2020) or intracranial volume (ICV) (Kelly et al., 2020, Monson et al., 2016, Thompson et al., 2016).

*Study participants were recruited from the same cohort as part of the Victorian Infant Brain Study (VIBeS) cohort between 2001 to 2004 (Kelly et al., 2016, Thompson et al., 2016, Thompson et al., 2020). There is the potential for overlap with cohorts in other studies (Kelly et al., 2020, Monson et al., 2016, Zhang et al., 2015) as participants were reported as being recruited from cohort studies conducted at the same hospital over the same recruitment period.

**Cohorts may overlap as study participants were recruited from the same hospital where prospective, longitudinal studies were ongoing (Vandewouw et al., 2019, Young et al., 2019).

*** Study participants were recruited from the same cohort as part of the Extremely Preterm Infants in Sweden Study (EXPRESS) between 2004 and 2007 (Broström et al., 2025, FernándezDeGamarra-Oca et al., 2024, Kvanta et al., 2023, Kvanta et al., 2024, Kvanta et al., 2021, Kvanta et al., 2025).

The mean GA at birth ranged from 25.0 to 34.6 weeks, with up to 138 participants classified as moderate to late preterm, 927 participants classified as very preterm, and 1003 participants classified as extremely preterm. The mean age at MRI assessment ranged from 6.4 to 10.5 years. Most preterm participants did not have any major chromosomal or genetic abnormalities. Age-matched term-born participants were included as a control group in each study, however, not all term-born children were recruited from birth. Children underwent MRI assessment in either a 1.5 Tesla or 3.0 Tesla MRI scanner and images were processed using automated software. There were five major outcome measures assessed across the studies: brain tissue volumes, cortical thickness, cortical surface area, white matter microstructure, and white matter connectivity. Eleven studies also reported data on the impact of perinatal factors on the brain, including perinatal brain injury, GA at birth, birthweight and being small for gestational age (SGA), patent ductus arteriosus (PDA) and bronchopulmonary dysplasia (ElMarroun et al., 2020, Kelly et al., 2020, Kelly et al., 2016, Kvanta et al., 2023, Monson et al., 2016, Thompson et al., 2022, Thompson et al., 2020, Thompson et al., 2016, Vandewouw et al., 2019, Young et al., 2019, Zubiaurre-Elorza et al., 2012). Additionally, seventeen studies reported data on neurodevelopmental outcomes, including cognition, motor function, visuospatial attention, and working memory (Broström et al., 2025, deKieviet et al., 2021, deKieviet et al., 2014, FernándezDeGamarra-Oca et al., 2024, Kelly et al., 2022, Kelly et al., 2024, Kelly et al., 2021, Kelly et al., 2016, Kvanta et al., 2025, Kvanta et al., 2024, Monson et al., 2016, Thompson et al., 2022, Thompson et al., 2019, Thompson et al., 2016, Tokariev et al., 2019, Young et al., 2019, Zubiaurre-Elorza et al., 2012).

### 3.1 Risk of bias analysis

**Fig. 2:**
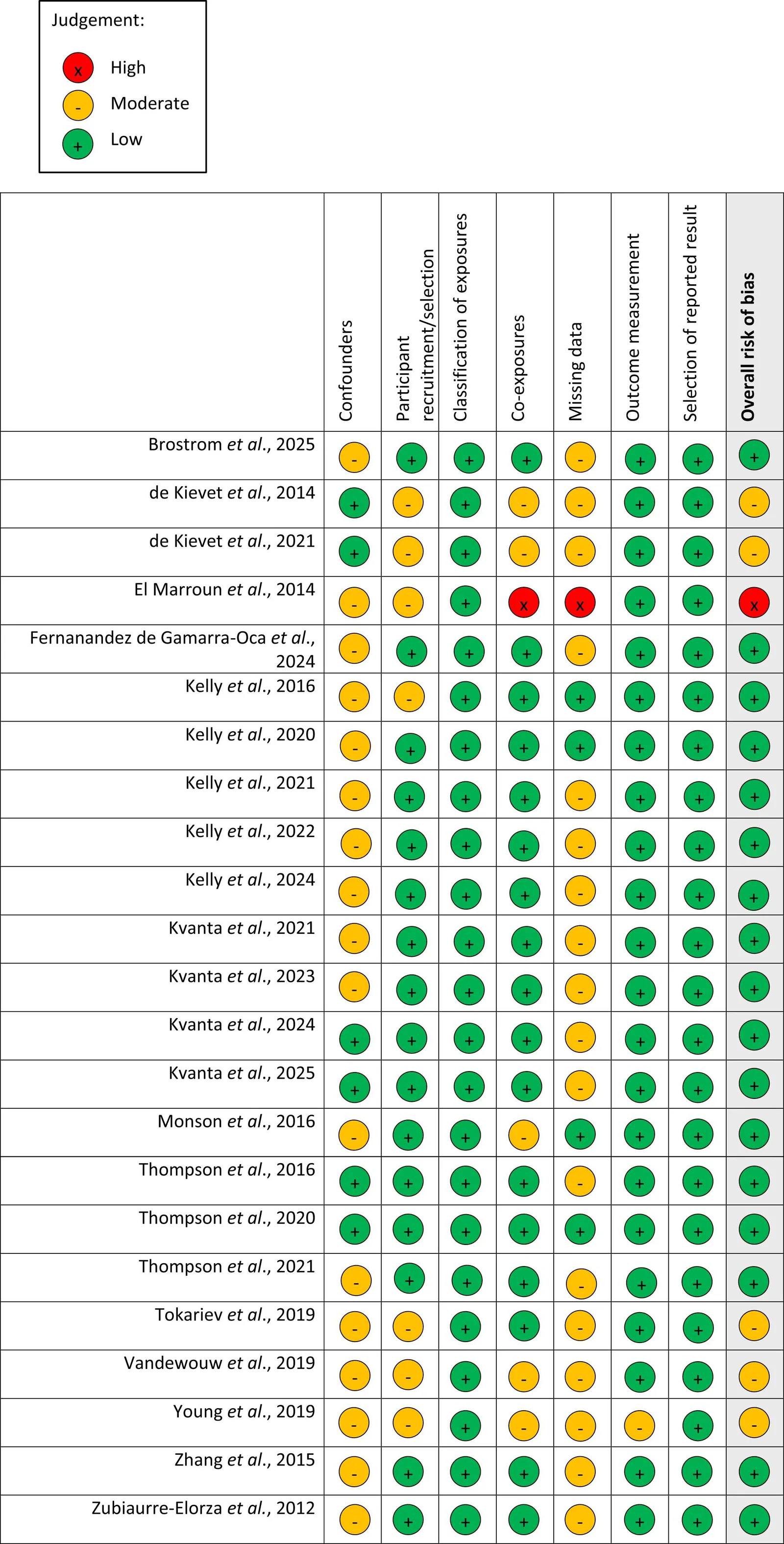
Summary of risk of bias analysis using ROBINS-E (Higgins et al., 2024).

Fig. 2 summarises the risk of bias judgements for all 23 studies. 22 studies had an overall low to moderate risk of bias rating: seventeen studies were judged as low risk (Broström et al., 2025, FernándezDeGamarra-Oca et al., 2024, Kelly et al., 2022, Kelly et al., 2024, Kelly et al., 2021, Kelly et al., 2020, Kelly et al., 2016, Kvanta et al., 2025, Kvanta et al., 2024, Kvanta et al., 2023, Kvanta et al., 2021, Monson et al., 2016, Thompson et al., 2022, Thompson et al., 2020, Thompson et al., 2016, Zhang et al., 2015, Zubiaurre-Elorza et al., 2012) and five were judged as moderate risk (deKieviet et al., 2021, deKieviet et al., 2014, Tokariev et al., 2019, Vandewouw et al., 2019, Young et al., 2019). One study was judged as having a high risk of bias overall due to a substantial loss of participants to follow up and reporting bias due to self-reported maternal data (ElMarroun et al., 2020). Of the twenty two studies that were judged as having an overall low to moderate risk of bias, Nineteen studies had a moderate or high risk of bias due to missing data (Broström et al., 2025, deKieviet et al., 2021, deKieviet et al., 2014, ElMarroun et al., 2020, FernándezDeGamarra-Oca et al., 2024, Kelly et al., 2022, Kelly et al., 2024, Kelly et al., 2021, Kvanta et al., 2025, Kvanta et al., 2024, Kvanta et al., 2023, Kvanta et al., 2021, Thompson et al., 2022, Thompson et al., 2016, Tokariev et al., 2019, Vandewouw et al., 2019, Young et al., 2019, Zhang et al., 2015, Zubiaurre-Elorza et al., 2012). This was the result of a loss of participants to follow up and/or the exclusion of participants due to low quality MRI data or an unsuccessful scan. Seventeen studies had a moderate risk of bias due to confounders resulting from a lack of adjustment for socioeconomic status and/or the inclusion of participants with white matter injury where it was not included as an independent variable (Broström et al., 2025, ElMarroun et al., 2020, FernándezDeGamarra-Oca et al., 2024, Kelly et al., 2022, Kelly et al., 2024, Kelly et al., 2021, Kelly et al., 2020, Kelly et al., 2016, Kvanta et al., 2023, Kvanta et al., 2021, Monson et al., 2016, Thompson et al., 2022, Tokariev et al., 2019, Vandewouw et al., 2019, Young et al., 2019, Zhang et al., 2015, Zubiaurre-Elorza et al., 2012).

### 3.2 Study methodology: MRI scanning procedures, motion correction, covariate adjustments, and corrections for multiple comparisons

All 23 studies included in this review reported MRI scanning protocols, with details including MRI machine make and model, MRI field strength, and image acquisition parameters.

Few studies reported on what participants were invited to do whilst in the scanner. Six studies reported that MRI scans were conducted without sedation (Kelly et al., 2024, Kvanta et al., 2023, Kvanta et al., 2021, Thompson et al., 2022, Thompson et al., 2019, Thompson et al., 2016). Tokariev et al. (Tokariev et al., 2019) conducted MRI scans in two sessions (one whilst watching a film) with a snack break in between to avoid child fatigue, and Young et al. (Young et al., 2019) conducted scans whilst the children were awake and watching a film. While the in-scanner task is not particularly relevant for interpreting differences in structural MRI measures, it helps to understand participant experience and efforts to reduce participant motion (Frew et al., 2022, Vanderwal et al., 2015).

Four studies reported that they provided participants with a mock MRI simulator session prior to commencing the scan, so that children could become familiar with the experience of completing an MRI scan (deKieviet et al., 2021, deKieviet et al., 2014, Kelly et al., 2016, Thompson et al., 2022). Nineteen studies reported data on motion correction methods used in their statistical analyses (Broström et al., 2025, deKieviet et al., 2021, deKieviet et al., 2014, ElMarroun et al., 2020, Kelly et al., 2022, Kelly et al., 2024, Kelly et al., 2021, Kelly et al., 2020, Kelly et al., 2016, Kvanta et al., 2025, Kvanta et al., 2024, Kvanta et al., 2023, Kvanta et al., 2021, Thompson et al., 2022, Thompson et al., 2020, Thompson et al., 2016, Tokariev et al., 2019, Vandewouw et al., 2019, Young et al., 2019). Six studies used tools within the Functional MRI of the Brain Software Library (FSL) for automated motion correction (deKieviet et al., 2021, deKieviet et al., 2014, Kelly et al., 2020, Kelly et al., 2016, Thompson et al., 2016, Young et al., 2019). For example, both Thompson et al. (Thompson et al., 2016) and de Kieviet et al. (deKieviet et al., 2021) used ‘eddy_correct’ to correct for eddy currents and participant motion artifacts, with Thompson et al. (Thompson et al., 2016) adding a weighting function to reduce bias caused by peripheral cerebrospinal fluid. Kelly et al. (Kelly et al., 2020) also used a variation of the FSL ‘eddy’ tool with slice-to-volume motion correction and then corrected for susceptibility-induced distortions using BrainSuite version 18. Fifteen studies reported visually inspecting and excluding poor quality MRI images prior to conducting their analyses (Broström et al., 2025, deKieviet et al., 2021, deKieviet et al., 2014, ElMarroun et al., 2020, Kelly et al., 2022, Kelly et al., 2024, Kelly et al., 2021, Kvanta et al., 2025, Kvanta et al., 2024, Kvanta et al., 2023, Kvanta et al., 2021, Thompson et al., 2022, Thompson et al., 2020, Vandewouw et al., 2019, Young et al., 2019).

There were also differences in adjustments and covariates included in analyses, across studies. Six studies adjusted for either total brain volume (deKieviet et al., 2014, ElMarroun et al., 2020, Thompson et al., 2020) or intracranial volume (Broström et al., 2025, Kelly et al., 2020, Kvanta et al., 2025, Kvanta et al., 2024, Kvanta et al., 2023, Kvanta et al., 2021, Monson et al., 2016, Thompson et al., 2016) in their MRI analyses. Three studies included maternal socioeconomic status (SES) as a covariate in their analyses (deKieviet et al., 2014, ElMarroun et al., 2020, Kvanta et al., 2025, Kvanta et al., 2024, Thompson et al., 2020) and another study performed a sensitivity analysis adjusting for maternal education level (Monson et al., 2016). In one study, where maternal SES was not included as a covariate or adjusted for, maternal education level was reportedly lower for preterm-born children than term-born children (Tokariev et al., 2019). In another study, El Marroun et al. (ElMarroun et al., 2020) found that in addition to lower maternal education, parental monthly income and the likelihood of having a partner were lower for preterm-born children than in term-born controls. However, a regression analysis that included maternal sociodemographic and lifestyle factors showed that these had no relationship with any observed brain differences (ElMarroun et al., 2020). Most studies found no significant difference in other demographic variables, including sex and age at MRI assessment, between preterm and term-born children (Broström et al., 2025, deKieviet et al., 2021, deKieviet et al., 2014, Kelly et al., 2022, Kelly et al., 2021, Kelly et al., 2020, Kvanta et al., 2025, Kvanta et al., 2024, Kvanta et al., 2023, Kvanta et al., 2021, Monson et al., 2016, Thompson et al., 2022, Vandewouw et al., 2019, Young et al., 2019, Zhang et al., 2015, Zubiaurre-Elorza et al., 2012).

Only eleven papers described correcting for multiple comparisons across multiple regions or cortical measures (Broström et al., 2025, ElMarroun et al., 2020, Kelly et al., 2024, Kelly et al., 2021, Kvanta et al., 2025, Kvanta et al., 2024, Kvanta et al., 2023, Kvanta et al., 2021, Monson et al., 2016, Thompson et al., 2022, Zhang et al., 2015).

### 3.3 Brain Tissue Volume

Several studies reported that preterm children had a smaller absolute brain volume (ElMarroun et al., 2020, Kelly et al., 2022, Kvanta et al., 2024, Monson et al., 2016, Vandewouw et al., 2019, Zhang et al., 2015) and subcortical grey matter volume than term-born children (ElMarroun et al., 2020, Kvanta et al., 2024, Kvanta et al., 2021, Monson et al., 2016). In particular, one study showed that tissue volumes were approximately 10% smaller in preterm children (Zhang et al., 2015). Studies also showed that preterm children had smaller cortical grey matter, white matter, and cerebellar volumes (Broström et al., 2025, deKieviet et al., 2014, ElMarroun et al., 2020, Kvanta et al., 2024, Kvanta et al., 2021, Zhang et al., 2015), and reduced volumes of the thalamus, hippocampus, basal ganglia, striatum, language-related regions, and motor network regions (Broström et al., 2025, deKieviet et al., 2014, Kvanta et al., 2024). There were also regional GM and WM volume differences (Kvanta et al., 2023).

Three studies reported data on longitudinal volumetric brain growth. Vandewouw et al. (Vandewouw et al., 2019) showed that between the ages of 4–12 years (measured at up to three different time points), white matter volume increased with age in both preterm and term-born groups, however the increase was larger in preterm children. In contrast, Thompson et al. (Thompson et al., 2020) did not find a significant difference in growth between groups across ages 7–13 years, before and after adjusting for total brain volume. However, paired *t*-tests showed that preterm children still had smaller total brain volumes at age 13 than term-born children. Within the same VIBeS cohort, there was a lower rate of increase in cortical volumes between term-corrected gestation and 7 years of age (Kelly et al., 2024).

### 3.4 Cortical thickness

Several studies found regional differences in cortical thickness when comparing preterm and term born cohorts. Cortical thickness was largely thinner in preterm children, across the whole brain (Kvanta et al., 2025), and particularly in the left postcentral gyrus, supramarginal gyrus, caudal middle rostral gyrus (Zubiaurre-Elorza et al., 2012), bilateral midcingulate cortex (Vandewouw et al., 2019), and language-related regions (Kvanta et al., 2024). However, one study indicated that cortical thickness was significantly thicker in preterm children in the bilateral occipital cortex, orbitofrontal cortex, bilateral inferior temporal and fusiform gyri, and the right inferior pre- and postcentral gyri (Vandewouw et al., 2019). The same study also reported data on longitudinal differences in cortical thickness for both preterm and term-born children. For both groups, cortical thickness significantly decreased with age across bilateral occipital, frontal, and temporal lobes. However, cortical thickness decreased more with age in preterm children, particularly in the left fusiform and inferior occipital gyri, and the right calcarine sulcus (Vandewouw et al., 2019). Longitudinally, cortical thickness was reduced at term-corrected gestation for preterm infants, with smaller increases from then until 7 years, resulting in similar or reduced regional cortical thickness (Kelly et al., 2024).

### 3.5 Cortical surface area

There was variation in results concerning cortical surface area between preterm and term-born children, across studies. Zhang et al. (2015) reported a larger cortical surface area in the precuneus and cingulate cortex whereas Vandewouw et al. (2019) reported that the left precuneus, posterior and middle cingulate cortex are smaller in preterm children than in term-born controls. Notably, the sample size was smaller in the study by Zhang et al. (2015) as it had only 48 participants in the final analysis compared to 266 in the study by Vandewouw et al. (2019). In the inferior orbital region, there was slower increase in cortical surface area between term-corrected gestation and 7 years (Kelly et al., 2024).

### 3.6 White Matter Microstructure

Seven studies reported that there were significant differences in white matter microstructure between preterm and term-born children (deKieviet et al., 2014, Kelly et al., 2021, Kelly et al., 2020, Kelly et al., 2016, Thompson et al., 2022, Tokariev et al., 2019, Young et al., 2019). Of these, three studies reported that FA was significantly lower in preterm children in structures such as the cingulum (deKieviet et al., 2014, Kelly et al., 2016, Young et al., 2019), inferior fronto-occipital tracts (deKieviet et al., 2014, Kelly et al., 2016), thalamocortical radiations and external capsule (Kelly et al., 2016, Young et al., 2019). Kelly et al. (2020) also showed that preterm children had significantly lower fibre density than term-born children, after adjusting for intracranial volume. There were also lower T1w/T2w values in the bilateral cingula (Thompson et al., 2022).

However, Tokariev et al. (2019) showed higher FA in preterm children compared to term-born controls in regions overlapping with the above, namely the inferior longitudinal fasciculus and inferior fronto-occipital fasciculus. Contrary to the other studies, this study did not identify any regions where FA was lower in preterm children than in term-born children. However, there was a large sample size discrepancy between the studies. The study by Tokariev et al. (2019) had the smallest sample size (n = 32) of the five studies reporting on differences in white matter microstructure.

Three studies identified differences in white matter diffusivity measures between groups; however, the evidence was conflicting. Tokariev et al. (2019) showed that MD and radial diffusivity RD) were lower in preterm children, particularly in the white matter tracts where FA was higher in preterm-born children, i.e., bilateral superior longitudinal fasciculus, inferior fronto-occipital fasciculus, inferior longitudinal fasciculus, corticospinal tract, inferior cerebellar peduncle. However, Young et al. (2019) reported that MD and RD were higher in preterm children in addition to the neurite orientation dispersion index, particularly in the corpus callosum, corona radiata, and left anterior limb of the internal and external capsules. Similarly, MD, RD, axial diffusivity (AD), and neurite orientation were higher in the fornices bilaterally in preterm infants, with lower neurite density in the left fornix, and higher neurite orientation dispersion in the left superior-lateral medial forebrain bundle (Kelly et al., 2021).

### 3.7 White matter connectivity

Two studies reported differences in white matter connectivity between preterm and term-born children (deKieviet et al., 2021, Thompson et al., 2016). Thompson et al. (Thompson et al., 2016) reported that preterm children had less dense connections, reduced integration, and higher segregation than term-born children, while small-worldness was similar between the groups. However, a later study reported that there was a higher degree of brain segregation and small-worldness in preterm children, and no significant difference in brain integration between the two groups (deKieviet et al., 2021).

### 3.8 Association with perinatal factors

#### 3.8.1 Perinatal brain injury

In total, eleven studies reported on the relationship between perinatal factors and differences in brain structure within preterm groups at school age. Many of these provided data on the influence of perinatal injury on the brain, particularly with respect to white matter injury. Preterm children with periventricular leukomalacia were shown to have a thicker cortex than preterm children without injury, specifically in the left rostral middle frontal region and right pericalcarine area (Zubiaurre-Elorza et al., 2012). Regional thickness differences, primarily increased thickness, were also seen with discrete white matter abnormalities (FernándezDeGamarra-Oca et al., 2024). The presence of grade I-II IVH was associated with small GM volume in the left hippocampus (Kvanta et al., 2023). The severity of injury also affected brain structure in preterm children. Three studies recorded the severity of whole brain abnormalities using the Kidokoro scoring system (Kelly et al., 2016, Kidokoro et al., 2013, Thompson et al., 2020, Thompson et al., 2016).

Thompson et al. (2020) reported that there was no association between neonatal moderate-to-severe brain abnormality and subsequent volumetric growth between the ages of 7 and 13 years, before or after adjusting for total brain volume. However, at the microstructural level, two studies associated a higher neonatal global brain abnormality score with reduced FA at age 7 (Kelly et al., 2016), and a lower fibre density and smaller increase in fibre density and cross-section between 7 and 13 years of age (Kelly et al., 2020). Thompson et al. (2016) also identified a weak association between neonatal brain abnormality and reduced connection density in the brain at age 7 years. As neonatal brain abnormality score increased, there was a decrease in connection strength in subnetworks involving structures of the basal ganglia, parietal cortex, pericalcarine and posterior cingulate cortices.

#### 3.8.2 Gestational age at birth

Four studies reported a correlation between GA at birth and brain tissue volumes, white matter microstructure, cortical surface area and structural connectivity in childhood (ElMarroun et al., 2020, Kelly et al., 2016, Thompson et al., 2016, Vandewouw et al., 2019). Longer gestation correlated with a larger total brain volume, grey matter, and white matter volumes in preterm 10-year-old children (ElMarroun et al., 2020). There was also evidence of a positive association between GA at birth and subcortical tissue volumes in the basal ganglia at age 10 years (ElMarroun et al., 2020). GA at birth correlated with FA within white matter tracts in 7-year-old children (Kelly et al., 2016). One study identified a positive correlation between GA at birth and cortical thickness in the right precuneus, cingulate cortex, and temporal gyri (Vandewouw et al., 2019). The same study also identified a negative correlation between GA at birth and cortical surface area; however, this association was specific to the superior and middle frontal gyri, middle temporal gyrus and superior temporal pole (Vandewouw et al., 2019). GA at birth also affected white matter connectivity in preterm children. A lower GA at birth was associated with lower density connections in the brain, reduced integration, higher segregation and increased small-worldness at age 7 years (Thompson et al., 2016).

#### 3.8.3 Birthweight

Thompson et al. (2020) reported that there was no significant difference in volumetric brain growth between ages 7–13 years in preterm children born SGA compared with preterm children born at an appropriate weight for their GA. However, after adjusting for total brain volume, there was evidence of poorer growth in the left insula in preterm children born SGA.

Three studies looked at the relationship between birthweight standard deviation (SD) scores and the impact on the brain in childhood (Kelly et al., 2016, Thompson et al., 2016). Birthweight SD scores are a comparative measure of birthweight relative to the mean weight expected of a child, adjusted for sex and age. This was calculated relative to the British Growth Reference (Cole et al., 1998). Kelly et al. (2016) found no evidence of a significant relationship between birthweight SD score and FA, axon dispersion or axon density in 7-year-old children. There was also no evidence of an association between birthweight SD score and global connectivity at age 7 years (Thompson et al., 2016). However, being SGA was associated with multiple lower T1w/T2w regional values at 7 years (Thompson et al., 2022).

#### 3.8.4 Bronchopulmonary dysplasia

After adjusting for total brain volume, bronchopulmonary dysplasia was not associated with less volumetric brain growth in preterm children between ages 7 and 13 years of age (Thompson et al., 2020), change in T1w/T2w values (Thompson et al., 2022), or global efficiency at age 7 years (Thompson et al., 2016). However, preterm children with bronchopulmonary dysplasia had reduced connection strength in parts of the left hemisphere, namely the inferior temporal cortex and its connections to both the lateral orbitofrontal cortex and amygdala (Thompson et al., 2016).

#### 3.8.5 Postnatal infection

Postnatal infection, defined as proven sepsis or necrotising enterocolitis, was not associated with T1w/T2w values or regional brain volumes at 7 years (Monson et al., 2016, Thompson et al., 2022). However, it was associated with reduced white matter connectivity (Thompson et al., 2016).

#### 3.8.6 Patent ductus arteriosus

One study described associations with PDA treatments; children with PDA requiring ligation was associated with smaller WM in the left frontal lobe, and larger GM in the occipital lobes, but no brain differences were seen in children with PDA requiring ibuprofen treatment only (Kvanta et al., 2023).

### 3.9 Neurodevelopmental correlations

In total, thirteen studies reported on the relationship between structural brain differences after preterm birth and neurodevelopmental outcomes (measured outside of the scanner) at school age (deKieviet et al., 2021, deKieviet et al., 2014, Kelly et al., 2022, Kelly et al., 2024, Kelly et al., 2021, Kelly et al., 2016, Monson et al., 2016, Thompson et al., 2022, Thompson et al., 2020, Thompson et al., 2016, Tokariev et al., 2019, Young et al., 2019, Zubiaurre-Elorza et al., 2012). Most studies estimated cognitive function by measures of verbal and non-verbal Intelligence Quotient (IQ), using a version of the Weschler Intelligence Scale (deKieviet et al., 2021, deKieviet et al., 2014, Kelly et al., 2016, Monson et al., 2016, Thompson et al., 2016, Young et al., 2019, Zubiaurre-Elorza et al., 2012). Thompson et al. (Thompson et al., 2020) assessed IQ at age 13 by Kaufman Brief Intelligence Test, 2nd Edition.

Children born preterm had lower total IQ scores than term-born children at age 7 years (deKieviet et al., 2021, Young et al., 2019) and total IQ correlated with grey and white matter volumes (deKieviet et al., 2014, Kvanta et al., 2024, Monson et al., 2016). This result was not significant when adjusting for intracranial volume (Monson et al., 2016). There was no correlation between volumetric growth between ages 7 and 13 and IQ at age 13 for children born preterm; however, volumetric growth positively correlated with IQ at age 13 for term-born children, particularly in the parietal lobe, frontal lobe, and limbic system (Thompson et al., 2020). Similarly, there was no correlation between IQ and longitudinal change in brain volumes from the neonatal period to 10 years (Kvanta et al., 2025). There were positive correlations between language skills and regional volumes of language areas, although these did not survive adjustment for IQ (Kvanta et al., 2024).

Two studies found a positive correlation between FA in white matter tracts and IQ in preterm children (Kelly et al., 2016, Young et al., 2019). By contrast, higher axial diffusivity, mean diffusivity and radial diffusivity were associated with lower IQ scores and visual motor integration in preterm children (Young et al., 2019). Preterm children with an atypically low IQ, defined as a score greater than one standard deviation below the normative mean, showed reduced connectivity predominantly on the right side of the brain (Thompson et al., 2016). This included the orbitofrontal cortices, cingulate cortex, parietal, temporal, and occipital lobes, thalamus, and hippocampus (Thompson et al., 2016). A faster increased in T1w/T2w regional values over childhood was also associated with higher IQ in all infants, and with attention, executive function, and mathematics in term children only (Thompson et al., 2022). There was no significant correlation between cortical thickness and IQ in children born preterm whereas cortical thickness negatively correlated with IQ in term-born children (Zubiaurre-Elorza et al., 2012). IQ and language scores were not associated with brain age delta (the difference between predicted and actual brain age) (Kelly et al., 2022), and IQ and visual spatial index only negatively correlated with mean right cortical thickness prior to correction for multiple comparisons (Kvanta et al., 2025). Smaller longitudinal increases in regional cortical thickness and cortical volumes between term-corrected gestation and 7 years were associated with impaired language (Kelly et al., 2024).

Eight studies assessed motor function outside of the scanner, mostly using the Movement Assessment Battery for Children to derive a total motor impairment score (deKieviet et al., 2021, deKieviet et al., 2014, FernándezDeGamarra-Oca et al., 2024, Kelly et al., 2016, Monson et al., 2016, Thompson et al., 2016). Young et al. (Young et al., 2019) assessed motor function using the Beery-Buktenica Test of Visual Motor Integration. Preterm children had poorer motor function compared to term-born controls at age 7.5 years (deKieviet et al., 2021, deKieviet et al., 2014). In preterm children, motor function positively correlated with white matter volume (deKieviet et al., 2014, Monson et al., 2016), cortical grey matter volume, and subcortical grey matter volume at age 7 (Monson et al., 2016), and basal ganglia, cerebellum, and motor execution and imagery network regions at 10 years (Broström et al., 2025). Motor function in preterm children also positively correlated with FA (deKieviet et al., 2014, Kelly et al., 2016) in several white matter tracts, including: corticospinal tract (deKieviet et al., 2014, Kelly et al., 2016), posterior limb of internal capsule, cerebral peduncle, corpus callosum, and corona radiata (Kelly et al., 2016), cingulum-hippocampal tract, forceps major and minor, and inferior fronto-occipital tract (deKieviet et al., 2014). One study showed that preterm children with impaired motor function, i.e., scoring more than one standard deviation below the normative mean, had reduced connection strength in a brain subnetwork involving the precuneus and its connections to the right inferior parietal lobe and right temporal lobe (Thompson et al., 2016). Motor scores were not associated with brain age delta (Kelly et al., 2022), or with cortical volume or thickness (FernándezDeGamarra-Oca et al., 2024).

Finally, four studies reported on memory skills. One study reported on the relationship between preterm birth, attention and working memory (Thompson et al., 2016) in 107 preterm children using the Test of Everyday Attention for Children and Backward Digital Recall subtest from the Working Memory Test Battery for Children (Pickering and Gathercole, 2001). There was no evidence for a significant correlation between white matter connectivity and attention or working memory. Similarly, memory was not associated with brain age delta (Kelly et al., 2022). However, improved episodic memory was associated with higher FA and lower MD/AD/RD in several regions (Kelly et al., 2021), and smaller increases in regional cortical thickness between term-corrected gestation and 7 years was associated with impaired memory (Kelly et al., 2024).

## 4 Discussion

This review suggests that whole brain MRI differences persist between preterm and term-born children at school age, and that these differences may correlate with cognitive and motor outcomes. In an age range where brain volume is relatively stable (Bethlehem et al., 2022), preterm children tend to have a smaller total brain volume and show significantly reduced grey and white matter volumes (deKieviet et al., 2014, ElMarroun et al., 2020, Kelly et al., 2022, Kvanta et al., 2021, Monson et al., 2016, Vandewouw et al., 2019, Zhang et al., 2015). The studies in this review did not find a clear association between perinatal brain injury and tissue volumes. In fact, volumetric differences were only evident in children without any significant perinatal complications. However, there was a clear positive correlation between brain tissue volumes and GA at birth in preterm children, such that children born later had larger brain tissue volumes (ElMarroun et al., 2020, Monson et al., 2016, Vandewouw et al., 2019, Zhang et al., 2015). While there is significant variability in neurocognitive outcomes across children, overall, this review supports prior evidence that at least some aspects of growth do not ‘catch up’ following preterm birth (Thompson et al., 2020).

Across studies, there was not evidence for whole brain cortical thickness and surface area differences as a function of preterm birth. Instead, evidence suggests that the associations are likely to be regional, occurring across several different locations in the brain across studies (FernándezDeGamarra-Oca et al., 2024, Vandewouw et al., 2019, Zhang et al., 2015, Zubiaurre-Elorza et al., 2012). This could be due to asynchronous brain development across different functional brain regions (corresponding to different functional roles/neurocognitive abilities; (Dubois et al., 2014)) and/or differences across children in the particular regions affected. There was also a lack of consensus on the direction of the correlation between preterm birth and cortical surface area, as two papers identified opposite relationships (Vandewouw et al., 2019, Zhang et al., 2015), with a further paper suggesting the relationship with surface area changed over time (Kelly et al., 2024). More studies are required to examine this relationship further and to reach a consensus.

The impact of prematurity on the brain also extends beyond the cortex. For example, preterm children show reduced FA in several white matter tracts, such as the corpus callosum, cingulum, inferior longitudinal fasciculus, and external capsule (deKieviet et al., 2014, Kelly et al., 2016, Young et al., 2019), which may correspond to neurodevelopmental outcomes, such as IQ (Kelly et al., 2016, Young et al., 2019) and motor function (Kelly et al., 2016). Reduced FA could suggest the presence of immature myelin and/or fewer myelinated axons in the brain or a reduced axon density and/or diameter (Hüppi and Dubois, 2006, Mori and Zhang, 2006). These white matter differences were also observed in preterm cohorts with perinatal brain injury and there was a positive association between white matter FA and GA at birth (Kelly et al., 2020, Kelly et al., 2016). However, one study (Tokariev et al., 2019) provides contradictory evidence, of higher FA in preterm children compared to term-born children, in regions associated with sensorimotor pathways. This study included infants born at < 28 weeks GA, without significant brain injury (Tokariev et al., 2019). This aligns with evidence that preterm infants can show regions of advanced brain development in the neonatal period due to the extrauterine stimulation of sensorimotor pathways (Giménez et al., 2008, Viola et al., 2011) and suggests these associations may persist into childhood.

Only two studies out of 23 provide data on whole-brain white matter connectivity. In both studies, brain segregation is higher in preterm children than in term-born children (deKieviet et al., 2021, Thompson et al., 2016). This result is consistent with prior research – not included in our review due to a lack of a term comparison group – that found that brain segregation was higher in extremely preterm children compared to moderately preterm children at school age (Fischi-Gómez et al., 2015). However, there was less evidence for true differences in integration and small-worldness. Thompson et al. (2016) also suggest a relationship between white matter connectivity and GA at birth, with a lower GA resulting in reduced integration, higher segregation and increased small-worldness. Research has shown that brain segregation tends to decrease with age, as long-range connections are favoured over shorter-range connections, representing more efficient information transfer in the brain (Grayson and Fair, 2017).

There was also little evidence of a correlation between birthweight or neonatal lung disease, such as bronchopulmonary dysplasia, and structural brain differences in preterm children at school age (Kelly et al., 2016, Thompson et al., 2020, Thompson et al., 2016). Bronchopulmonary dysplasia can have adverse associations with cerebral white matter volumes in preterm infants (Lee et al., 2019), yet based on the two studies reporting on this in the current review, there were no differences in volumetric brain growth or global measures of white matter connectivity at school age. These perinatal factors represent the limited evidence in the literature pertaining to associations between risk or protective factors and brain differences in preterm-born school-aged children. This is a key area where research is needed to understand how children could be supported to optimise their outcomes.

The included studies showed variation in the relationship between structural brain differences in school-age children born preterm and developmental outcomes. While there was some evidence for an association between cognitive development and grey matter volume, this association varied depending on adjustments – i.e., controlling for possible confounding variables (deKieviet et al., 2014, Kvanta et al., 2024, Monson et al., 2016); there were also varied associations between cognitive development and regional brain growth (Thompson et al., 2020). Similarly, associations between white matter microstructure/connectivity and cognitive development and memory varied by region/tract and white matter metric (Kelly et al., 2021, Kelly et al., 2016, Thompson et al., 2022, Thompson et al., 2016, Young et al., 2019). We observed more consistent associations between motor function and a range of brain metrics (Broström et al., 2025, deKieviet et al., 2021, deKieviet et al., 2014, Kelly et al., 2016, Monson et al., 2016, Thompson et al., 2016). The relationship between structural brain metrics and cognitive outcomes in school-age preterm-born children is nuanced, with need for well-powered, hypothesis-driven studies to identify robust associations.

Given that preterm birth appears causally related to developmental delay (Zambrana et al., 2021), understanding how brain differences contribute to possible mediation of these outcomes is crucial. Future research should aim to clarify this potential mediation through longitudinal studies with multimodal brain imaging and long-term outcome assessments.

### 4.1 Strengths and limitations

All 23 studies included in this review had a prospective, longitudinal design which was appropriate for assessing significant changes over time, and 22 of 23 studies were judged low or moderate risk of bias. However, there are important limitations of the reviewed research that make it difficult to draw broad conclusions.

First, the reviewed research is that cohort sizes were considerably different between studies (i.e., eleven studies >100 preterm children and seven studies with <40 preterm children), which raises the risk of conflicting results due to underpowered analyses and type II errors in the smaller studies. Smaller samples were often the result of a loss of participants to follow up – something that might have meant fewer children with developmental difficulties taking part, since completing an MRI scan requires staying still for an extended period of time. A further complication is that several studies were likely to have had overlapping (i.e. non-independent) cohorts, as participants were selected from the same hospital (e.g., ten studies from the Royal Women’s Hospital, Melbourne).

Second, there was limited adjustment across studies for other factors that could affect results, such as gestational age bracket, or maternal socioeconomic status (SES). Only four studies reported correlations with gestational age, limiting our capacity to examine variability between sub-groups in degree of prematurity. While lower maternal SES has been associated with poorer cognitive outcomes for children born preterm (Joseph et al., 2018, Wong and Edwards, 2013), only six of the 23 studies reported an adjustment for SES. It is also unclear whether samples were culturally diverse as there was limited reporting of this characteristic across the studies included in this review. Therefore, additional research is necessary to determine whether reported associations are generalisable. The variation in whether results were adjusted for total brain volume, intracranial volume, or not, also makes comparing results challenging. All three approaches could be appropriate to answer specific questions in preterm infants, but this needs justification.

A third limitation concerns the variability of tools and methodological decisions across studies. For example, some studies used MRI scanners with reduced magnetic field strengths (i.e., two studies with 1.5 T MRI; twenty one studies with 3 T MRI). This is important as a higher field strength results in faster imaging times and vastly improved spatial and temporal resolution, which improve the quality of the images and enables the observation of smaller structures in the child brain (Dagia and Ditchfield, 2008). Equally, not all studies reported motion correction procedures in their methodology or outlined clearly whether they had matched data quality between preterm and term-born groups; studies also implemented a variety of approaches to motion correction. Data quality and participant motion are important to consider as they can both obscure true effects and create spurious false positive results. Participant motion can also overestimate the relationship between age and brain connectivity, as motion tends to decrease with age (Grayson and Fair, 2017, Satterthwaite et al., 2012). Definition of the term-born control children may also contribute to study findings. The articles included here commonly defined this as ≥ 37 weeks’ GA (where specified), but there is known variation in brain structure between 37 and 42 weeks of birth GA (ElMarroun et al., 2020).

Across studies, there were a range of approaches taken to correct for multiple comparisons in whole-brain analyses, with some papers not describing any approach. This may reflect a lack of consensus in the field for when such corrections are necessary; confirmatory studies replicating specific relationships will be important to determine robustness.

In sum, lack of consensus between studies could arise from the prevalence of inadequately powered studies and the use of inferior imaging technology and protocols. Recent moves towards open-source, automated analysis pipelines for motion correction (Gorgolewski et al., 2011), multi-site collaborations for acquiring large, longitudinal datasets (e.g. (Volkow et al., 2024)), as well as pre-registration of analysis decisions and sharing of protocols (e.g., via the Open Science Framework) should increase both the quality and transparency of research moving forward.

One limitation of the present review was the exclusion of functional brain differences between preterm and term-born children at school age. A recent review has also highlighted how MRI can be used to investigate structure-function relationships in neurological disorders, including white matter injury (Fotiadis et al., 2024). While out of the scope of the present review, future efforts should be made to explore parallels between the structural connectome and the dynamics of functional connectivity in childhood brain development following preterm birth.

Finally, recent reviews have begun to study the impact of emerging neuroprotective strategies in the postnatal period, for instance music therapy, on brain development following preterm birth in infants (Mohan et al., 2021, Yue et al., 2021). It has been shown that early exposure to the extrauterine environment can adversely affect brain development, and this can be exacerbated by over-exposure or underexposure to noise in the NICU environment itself (Haslbeck and Bassler, 2018, Pineda et al., 2014). Therefore, exploring methods of controlled postnatal auditory stimuli could call attention to a low cost and non-invasive therapy that could support neurodevelopment in children born prematurely (Lordier et al., 2019). It will be important for future research and reviews to explore the longer-term impacts of these strategies.

## 5 Conclusion

Preterm birth is associated with significant whole brain MRI differences at school age, specifically reduced tissue volumes and aberrant white matter microstructure. These differences were influenced by perinatal factors such as a lower GA at birth and the incidence of perinatal brain injury. This review has both contributed to the existing published literature and highlighted the lack of data available from longitudinal cohort studies that specifically set out to determine the impact of preterm birth on the neuroanatomy in school-aged children. Increased publication of study protocols and improved data sharing would undoubtedly benefit researchers focused on exploring the long-term effects of preterm birth on the brain. Not only would this enable the reproducibility of results, but it would also provide the opportunity to conduct mega-analyses to establish trends. It would be of further interest to ascertain whether any specific perinatal therapies, such as music therapy in neonatal units, have beneficial or adverse effects on brain development in later childhood. This may provide evidence in favour of the use of early neuroprotective strategies, or rather provide the impetus for introducing new ones.

## Data Statement

The authors confirm that the data supporting the findings of this study are available within the article and its supplementary material.

## CRediT authorship contribution statement

**Ebony Coward:** Writing – original draft, Visualization, Methodology, Formal analysis, Conceptualization. **Katie Mckinnon:** Formal analysis, Investigation, Visualization, Writing – review & editing. **Hilary Richardson:** Writing – review & editing, Supervision, Formal analysis. **Sue Fletcher-Watson:** Writing – review & editing, Supervision, Project administration, Methodology, Conceptualization.

## Funding

This work was supported by the 10.13039/100004440Wellcome Trust [grant number 218493/Z/19/Z].

## Declaration of Competing Interest

The authors declare that they have no known competing financial interests or personal relationships that could have appeared to influence the work reported in this paper.

## Data Availability

The authors confirm that the data supporting the findings of this study are available within the article and its supplementary material.

## Glossary

**Fractional anisotropy**: A measure of directional water displacement used to characterise white matter microstructure in the brain. It measures the asymmetrical dispersion of water molecules when impeded by tissues, such as white matter bundles.
**Mean diffusivity**: A measure of the average water dispersal associated with axon orientation and myelination.
**Axial diffusivity**: A measure of directional water displacement that measures water dispersion in parallel to the white matter tracts being investigated.
**Radial diffusivity**: A measure of directional water displacement that measures water dispersion perpendicular to the white matter tracts being investigated.
**Integration**: A measure of white matter connectivity that refers to the brain’s ability to combine information from widely distributed brain regions, i.e., a ‘global’ approach to interpreting information.
**Segregation**: A measure of white matter connectivity that describes the processing of information within densely interconnected regions of the brain, i.e., a more ‘local’, clustered approach.
**Small-worldness**: Describes a network of white matter connectivity which favours short-range connections between different areas of the brain, and a high degree of clustering. (Kidokoro et al., 2013)(Kidokoro et al., 2013)(Kidokoro et al., 2013)
**Kidokoro Scoring System**: This system involves assigning a score (0−4) to regional tissue abnormalities observed on neonatal images, such as focal signal abnormalities, delayed myelination, thinning of the corpus callosum and reduced white matter volume. A composite global brain abnormality score is derived and classified as normal (total score 0–3), mild (total score 4–7), moderate (total score 8–11), or severe (total score 12) (Kidokoro et al., 2013).
