# Supplementary material for: A systematic review of structural brain differences in school-aged children born preterm
Source: Dev Cogn Neurosci. 2026 Sep 10;81:101818. doi: 10.1016/j.dcn.2026.101818 (PMC13594853; doi:10.1016/j.dcn.2026.101818)
Supplement: Supplementary file 1 [file mmc1.docx]

**Supplement – search strategy**

Database: Embase <1974 to 2021 February 03>, Ovid MEDLINE(R) and Epub Ahead of Print, InProcess & Other Non-Indexed Citations, Daily and Versions(R) <1946 to February 01, 2021> Search Strategy:

--------------------------------------------------------------------------------

**SEARCH up to 15/02/2021**

exp premature labor/ or "immature and premature labor"/ (74488)

exp prematurity/ or "immature and premature labor"/ or newborn disease/ (125684)

preterm.mp. (186307)

premature.mp. (406840)

gestational age/ (224718)

1 or 2 or 3 or 4 or 5 (693483)

nuclear magnetic resonance imaging/ or diffusion tensor imaging/ or diffusion weighted

imaging/ or dynamic contrast-enhanced magnetic resonance imaging/ (869938)

neuroimaging/ or neurologic examination/ (235186)

MRI.mp. (726033)

magnetic resonance imag*.mp. (1497565)

diffusion weighted image*.mp. (6276)

diffusion tensor image*.mp. (1478)

7 or 8 or 9 or 10 or 11 or 12 (1739361)

6 and 13 (23263)

child/ or juvenile/ or school child/ (3662754)

child development/ (92987)

15 or 16 (3704319)

14 and 17 (4269)

cohort analysis/ (942423)

comparative study/ (2767419)

controlled study/ or case control study/ (8338576)

longitudinal study/ or clinical study/ (451855)

prospective study/ (1222543)

follow up/ (1642374)

outcome*.mp. (6164764)

cohort*.mp. (1958835)

brain/ or gray matter/ or white matter/ (907715)

exp brain development/ (47127)

neuroanatomy/ (28319)

brain size/ (31882)

brain structure.mp. (18576)

neurodevelopment*.mp. (89169)

connectivity.mp. (142789)

connection*.mp. (326269)

exp nerve tract/ (59854)

19 or 20 or 21 or 22 or 23 or 24 or 25 or 26 or 27 or 28 or 29 or 30 or 31 or 32 or 33 or 34 or

35 (18584669)

18 and 36 (3191)

37 not (Animals/ not (Animals/ and Humans/)) (3191)

limit 38 to yr="2006 -Current" (2786)

Premature Birth/ (63504)

exp Infant, Premature/ (163524)

exp Infant, Premature, Diseases/ (151160)

preterm birth.mp. (45796)

preterm.mp. (186307)

prematur*.mp. (518137)

40 or 41 or 42 or 43 or 44 or 45 (573998)

magnetic resonance imaging/ or exp diffusion magnetic resonance imaging/ (974082)

MRI.mp. (726033)

magnetic resonance image$.mp. (31099)

diffusion tensor image$.mp. (1478)

neuroimaging/ or diffusion tensor imaging/ or neuroradiography/ (190150)

diffusion weighted imag*.mp. (61685)

47 or 48 or 49 or 50 or 51 or 52 (1417682)

46 and 53 (12649)

Child/ (3500277)

Child Development/ (92987)

child*.mp. (5264604)

school child*.mp. (415823)

55 or 56 or 57 or 58 (5264604)

54 and 59 (4345)

cohort studies/ or follow-up studies/ or longitudinal studies/ or prospective studies/ (3388624)

outcome$.mp. (6164764)

follow-up.mp. (3456606)

cohort$.mp. (1958835)

brain/ or gray matter/ or white matter/ (907715)

volume$.mp. (2040364)

brain development.mp. (72633)

brain structure.mp. (18576)

neurodevelopment*.mp. (89169)

exp Neural Pathways/ (171639)

connectivity.mp. (142789)

connection$.mp. (326269)

61 or 62 or 63 or 64 or 65 or 66 or 67 or 68 or 69 or 70 or 71 or 72 (12632822)

60 and 73 (3215)

74 not (Animals/ not (Animals/ and Humans/)) (3209)

limit 75 to yr="2006 -Current" (2788)

39 or 76 (4003)

remove duplicates from 77 (3235)

--------------------------------------------------------------------------------

**SEARCH up to 15/12/2025**

| 1 | exp premature labor/ or "immature and premature labor"/ | 327,090 |
| --- | --- | --- |
| 2 | limit 1 to yr="2021 -Current" | 91,971 |
| 3 | exp prematurity/ or "immature and premature labor"/ or newborn disease/ | 243,884 |
| 4 | limit 2 to yr="2021 -Current" | 91,971 |
| 5 | preterm.mp. | 508,965 |
| 6 | limit 5 to yr="2021 -Current" | 139,763 |
| 7 | premature.mp. | 1,039,407 |
| 8 | limit 7 to yr="2021 -Current" | 224,044 |
| 9 | gestational age/ | 380,100 |
| 10 | limit 9 to yr="2021 -Current" | 102,787 |
| 11 | 2 or 4 or 6 or 8 or 10 | 379,198 |
| 12 | nuclear magnetic resonance imaging/ or diffusion tensor imaging/ or diffusion weighted imaging/ or dynamic contrast-enhanced magnetic resonance imaging/ | 1,633,723 |
| 13 | limit 12 to yr="2021 -Current" | 488,488 |
| 14 | neuroimaging/ or neurologic examination/ | 431,119 |
| 15 | limit 14 to yr="2021 -Current" | 127,695 |
| 16 | MRI.mp. | 2,076,254 |
| 17 | limit 16 to yr="2021 -Current" | 609,831 |
| 18 | magnetic resonance imag*.mp. | 3,118,311 |
| 19 | limit 18 to yr="2021 -Current" | 844,537 |
| 20 | diffusion weighted image*.mp. | 24,558 |
| 21 | limit 20 to yr="2021 -Current" | 5,851 |
| 22 | diffusion tensor image*.mp. | 4,547 |
| 23 | limit 22 to yr="2021 -Current" | 1,466 |
| 24 | 13 or 15 or 17 or 19 or 21 or 23 | 1,058,143 |
| 25 | 11 and 24 | 28,335 |
| 26 | child/ or juvenile/ or school child/ | 5,480,655 |
| 27 | limit 26 to yr="2021 -Current" | 1,184,783 |
| 28 | child development/ | 130,340 |
| 29 | limit 28 to yr="2021 -Current" | 20,048 |
| 30 | 27 or 29 | 1,191,997 |
| 31 | 25 and 30 | 3,073 |
| 32 | cohort analysis/ | 2,304,885 |
| 33 | limit 32 to yr="2021 -Current" | 1,118,483 |
| 34 | comparative study/ | 3,324,767 |
| 35 | limit 34 to yr="2021 -Current" | 404,743 |
| 36 | controlled study/ or case control study/ | 15,115,166 |
| 37 | limit 36 to yr="2021 -Current" | 4,969,268 |
| 38 | longitudinal study/ or clinical study/ | 793,894 |
| 39 | limit 38 to yr="2021 -Current" | 224,206 |
| 40 | prospective study/ | 2,044,798 |
| 41 | limit 40 to yr="2021 -Current" | 610,056 |
| 42 | follow up/ | 3,267,286 |
| 43 | limit 42 to yr="2021 -Current" | 1,115,435 |
| 44 | outcome*.mp. | 15,659,444 |
| 45 | limit 44 to yr="2021 -Current" | 4,894,274 |
| 46 | cohort*.mp. | 6,103,427 |
| 47 | limit 46 to yr="2021 -Current" | 2,403,628 |
| 48 | brain/ or gray matter/ or white matter/ | 1,171,699 |
| 49 | limit 48 to yr="2021 -Current" | 190,814 |
| 50 | exp brain development/ | 72,602 |
| 51 | limit 50 to yr="2021 -Current" | 16,700 |
| 52 | neuroanatomy/ | 42,105 |
| 53 | limit 52 to yr="2021 -Current" | 6,886 |
| 54 | brain size/ | 60,671 |
| 55 | limit 54 to yr="2021 -Current" | 17,554 |
| 56 | brain structure.mp. | 66,920 |
| 57 | limit 56 to yr="2021 -Current" | 22,457 |
| 58 | neurodevelopment*.mp. | 296,858 |
| 59 | limit 58 to yr="2021 -Current" | 123,515 |
| 60 | connectivity.mp. | 414,953 |
| 61 | limit 60 to yr="2021 -Current" | 161,060 |
| 62 | connection*.mp. | 1,196,165 |
| 63 | limit 62 to yr="2021 -Current" | 367,422 |
| 64 | exp nerve tract/ | 84,257 |
| 65 | limit 64 to yr="2021 -Current" | 15,799 |
| 66 | 33 or 35 or 37 or 39 or 41 or 43 or 45 or 47 or 49 or 51 or 53 or 55 or 57 or 59 or 61 or 63 or 65 | 10,402,199 |
| 67 | 31 and 66 | 2,461 |
| 68 | 67 not (Animals/ not (Animals/ and Humans/)) | 2,461 |
| 69 | Premature Birth/ | 199,081 |
| 70 | limit 69 to yr="2021 -Current" | 72,690 |
| 71 | exp Infant, Premature/ | 286,521 |
| 72 | limit 71 to yr="2021 -Current" | 77,467 |
| 73 | exp Infant, Premature, Diseases/ | 266,949 |
| 74 | limit 73 to yr="2021 -Current" | 70,984 |
| 75 | preterm birth.mp. | 152,832 |
| 76 | limit 75 to yr="2021 -Current" | 53,844 |
| 77 | preterm.mp. | 508,965 |
| 78 | limit 77 to yr="2021 -Current" | 139,763 |
| 79 | prematur*.mp. | 1,341,553 |
| 80 | limit 79 to yr="2021 -Current" | 299,543 |
| 81 | 70 or 72 or 74 or 76 or 78 or 80 | 347,377 |
| 82 | magnetic resonance imaging/ or exp diffusion magnetic resonance imaging/ | 2,058,154 |
| 83 | limit 82 to yr="2021 -Current" | 580,367 |
| 84 | MRI.mp. | 2,076,254 |
| 85 | limit 84 to yr="2021 -Current" | 609,831 |
| 86 | magnetic resonance image$.mp. | 93,483 |
| 87 | limit 86 to yr="2021 -Current" | 16,182 |
| 88 | diffusion tensor image$.mp. | 4,547 |
| 89 | limit 88 to yr="2021 -Current" | 1,466 |
| 90 | neuroimaging/ or diffusion tensor imaging/ or neuroradiography/ | 367,999 |
| 91 | limit 90 to yr="2021 -Current" | 119,280 |
| 92 | diffusion weighted imag*.mp. | 169,192 |
| 93 | limit 92 to yr="2021 -Current" | 58,244 |
| 94 | 83 or 85 or 87 or 89 or 91 or 93 | 936,049 |
| 95 | 81 and 94 | 20,722 |
| 96 | Child/ | 5,245,464 |
| 97 | limit 96 to yr="2021 -Current" | 1,161,335 |
| 98 | Child Development/ | 130,340 |
| 99 | limit 98 to yr="2021 -Current" | 20,048 |
| 100 | child*.mp. | 10,812,523 |
| 101 | limit 100 to yr="2021 -Current" | 2,160,956 |
| 102 | school child*.mp. | 735,089 |
| 103 | limit 102 to yr="2021 -Current" | 153,878 |
| 104 | 97 or 99 or 101 or 103 | 2,160,956 |
| 105 | 95 and 104 | 10,123 |
| 106 | cohort studies/ or follow-up studies/ or longitudinal studies/ or prospective studies/ | 7,216,327 |
| 107 | limit 106 to yr="2021 -Current" | 2,481,255 |
| 108 | outcome$.mp. | 15,659,444 |
| 109 | limit 108 to yr="2021 -Current" | 4,894,274 |
| 110 | follow-up.mp. | 9,020,549 |
| 111 | limit 110 to yr="2021 -Current" | 2,416,572 |
| 112 | cohort$.mp. | 6,103,427 |
| 113 | limit 112 to yr="2021 -Current" | 2,403,628 |
| 114 | brain/ or gray matter/ or white matter/ | 1,171,699 |
| 115 | limit 114 to yr="2021 -Current" | 190,814 |
| 116 | volume$.mp. | 18,024,660 |
| 117 | limit 116 to yr="2021 -Current" | 3,092,980 |
| 118 | brain development.mp. | 167,081 |
| 119 | limit 118 to yr="2021 -Current" | 43,015 |
| 120 | brain structure.mp. | 66,920 |
| 121 | limit 120 to yr="2021 -Current" | 22,457 |
| 122 | neurodevelopment*.mp. | 296,858 |
| 123 | limit 122 to yr="2021 -Current" | 123,515 |
| 124 | exp Neural Pathways/ | 205,812 |
| 125 | limit 124 to yr="2021 -Current" | 24,030 |
| 126 | connectivity.mp. | 414,953 |
| 127 | limit 126 to yr="2021 -Current" | 161,060 |
| 128 | connection$.mp. | 1,196,165 |
| 129 | limit 128 to yr="2021 -Current" | 367,422 |
| 130 | 107 or 109 or 111 or 113 or 115 or 117 or 119 or 121 or 123 or 125 or 127 or 129 | 8,826,275 |
| 131 | 105 and 130 | 9,549 |
| 132 | 131 not (Animals/ not (Animals/ and Humans/)) | 2,362 |
| 133 | 68 or 132 | 3,328 |
| 134 | remove duplicates from 133 | 2,360 |

**COMBINED** - 5595
